# Anticancer activity of a novel small molecule tubulin inhibitor STK899704

**DOI:** 10.1371/journal.pone.0173311

**Published:** 2017-03-15

**Authors:** Krisada Sakchaisri, Sun-Ok Kim, Joonsung Hwang, Nak Kyun Soung, Kyung Ho Lee, Tae Woong Choi, Yongjun Lee, Chan-Mi Park, Naraganahalli R. Thimmegowda, Phil Young Lee, Bettaswamigowda Shwetha, Ganipisetti Srinivasrao, Thi Thu Huong Pham, Jae-Hyuk Jang, Hye-Won Yum, Young-Joon Surh, Kyung S. Lee, Hwangseo Park, Seung Jun Kim, Yong Tae Kwon, Jong Seog Ahn, Bo Yeon Kim

**Affiliations:** 1 Anticancer Agents Research Center, Korea Research Institute of Bioscience and Biotechnology (KRIBB), Ochang, Cheongwon, Korea; 2 Department of Pharmacology, Faculty of Pharmacy, Mahidol University, Bangkok, Thailand; 3 The Key Laboratory of Enzyme & Protein Technology (KLEPT), VNU University of Science, Vietnam National University, Hanoi, Vietnam; 4 College of Pharmacy, Seoul National University, Seoul, Korea; 5 Laboratory of Metabolism, National Cancer Institute, National Institutes of Health, Bethesda, Maryland, United States of America; 6 Department of Bioscience and Biotechnology, Sejong University, Seoul, Korea; 7 Disease Target Structure Research Center, Korea Research Institute of Bioscience and Biotechnology, Daejeon, Korea; 8 Department of Biomedical Sciences and Protein Metabolism Medical Research Center, College of Medicine, Seoul National University, Seoul, Korea; Columbia University, UNITED STATES

## Abstract

We have identified the small molecule STK899704 as a structurally novel tubulin inhibitor. STK899704 suppressed the proliferation of cancer cell lines from various origins with IC_50_ values ranging from 0.2 to 1.0 μM. STK899704 prevented the polymerization of purified tubulin *in vitro* and also depolymerized microtubule in cultured cells leading to mitotic arrest, associated with increased Cdc25C phosphorylation and the accumulation of both cyclin B1 and polo-like kinase 1 (Plk1), and apoptosis. Unlike many anticancer drugs such as Taxol and doxorubicin, STK899704 effectively displayed antiproliferative activity against multidrug-resistant cancer cell lines. The proposed binding mode of STK899704 is at the interface between αβ-tubulin heterodimer overlapping with the colchicine-binding site. Our *in vivo* carcinogenesis model further showed that STK 899704 is potent in both the prevention and regression of tumors, remarkably reducing the number and volume of skin tumor by STK899704 treatment. Moreover, it was significant to note that the efficacy of STK899704 was surprisingly comparable to 5-fluorouracil, a widely used anticancer therapeutic. Thus, our results demonstrate the potential of STK899704 to be developed as an anticancer chemotherapeutic and an alternative candidate for existing therapies.

## Introduction

Microtubules, a major component of the cytoskeleton, are polymers of α- and β-tubulin heterodimers which play important roles in a variety of cellular processes including cellular trafficking, maintaining cell polarity, cell signaling, cell migration, and cell proliferation [1]. During mitosis, microtubules form highly dynamic mitotic spindles, which are critical for the proper orientation and segregation of chromosomes [2]. The impairment of mitotic spindles leads to mitotic arrest and consequently apoptosis [3, 4]. The critical role of microtubules in cell division and other cellular functions makes them an attractive target for cancer chemotherapy.

Microtubule-targeting agents are usually classified into two main groups, stabilizers and destabilizers, based on their mechanisms of action [5–7]. Microtubule-stabilizing agents, including paclitaxel (Taxol) and docetaxel, inhibit depolymerization and enhance microtubule polymerization. Most microtubule-stabilizing agents bind to the taxane-binding site or an overlapping site on β-tubulin. Microtubule-depolymerizing agents such as colchicine and vinca alkaloids, inhibit microtubule polymerization and usually bind to either the colchicine- or vinca-binding site. Both stabilizers and destabilizers affect microtubule dynamics at lower concentrations than those that affect microtubule-polymer mass [5], and arrest cells at mitosis. Although microtubule-targeting agents especially paclitaxel and vinca alkaloids are widely used and in clinical success, both intrinsic and acquired drug resistances in cancer cells are significant limitations to clinical efficacy [8–10]. Resistance to microtubule-targeting agents is often related to the expression of multidrug resistance proteins such as the drug efflux pump P-glycoprotein (P-gp), resulting in the exportation of the agents from cancer cells preventing the intracellular accumulation of the active drug. Resistance can also arise from mutations in and/or alteration of tubulin isotype levels [11, 12]. In addition to drug resistance, neurotoxicity is a common side effect, which leads to a dose limitation of microtubule targeting drugs in clinical use [13, 14]. Therefore, in recent years, there has been great interest in the identification of novel tubulin-targeting drugs with lowered neurotoxicity and insensitivity to chemoresistance providing significant clinical benefits to cancer patients.

In our screening for antiproliferative agents from a small-molecule library, we identified STK899704 as a structurally novel antimitotic agent. STK899704 binds tubulin and inhibits its polymerization, leading to cell cycle arrest at mitosis and cell death. Molecular docking studies demonstrated that the binding site of STK899704 on tubulin overlaps with the colchicine-binding site. In addition, STK899704 exhibited antiproliferative activity against a broad range of cancer cell types regardless of multidrug-resistance phenotypes. The preclinical evaluation of novel compounds STK899704 revealed the effect on skin carcinogenesis model *in vivo*, demonstrating its chemopreventive and antitumor activities. As far as we know, this is the first study indicating that STK899704 has a potent therapeutic efficacy. Thus, our data suggest that STK899704 is a novel tubulin-depolymerizing agent with the potential to be further developed as an anticancer agent.

## Materials and methods

### Reagents, antibodies, cell lines, and animals

Dulbecco’s modified Eagle’s medium (DMEM) and RPMI 1640 were purchased from HyClone. Fetal bovine serum (FBS), McCoy’s 5a medium, and Alexa Fluor 488 conjugated α-tubulin antibody were supplied by Invitrogen. Doxorubicin, paclitaxel, nocodazole, Hoechst 33342, DMBA (7,12-dimethylbenz[α]anthracene), TPA (12-O-tetradecanoylphorbol-13-acetate), 5-fluorouracil (5-FU), and antibodies against β-actin and γ-actin were obtained from Sigma-Aldrich. Vinblastine and colchicine were purchased from Calbiochem/EMD Chemicals. All compounds were dissolved in DMSO at a 0.1% final concentration for *in vitro* analyses. Antibodies against phospho-Histone H3 (S10), Histone H3, Cyclin B1, caspase-8, and caspase-9 were supplied by Cell Signaling Technology. The Caspase-3 antibody was obtained from IMGENEX. Antibodies against Cdc25C, Plk1, caspase-7, PARP, and GAPDH were purchased from Santa Cruz Biotechnology. Z-VAD-FMK was obtained from R&D Systems. STK899704 was synthesized at Korea Research Institute of Bioscience and Biotechnology (KRIBB), and both detailed synthetic procedure and characterization data are shown in S1 Fig. All the intermediates and final compound were characterized by NMR and ESIMS analyses. Analytical data of STK899704 as follows.

^**1**^**H NMR** (400 MHz, DMSO-*d*_*6*_) δ ppm:11.838 (1H, s, CH_3_-NH-CO-CH_3_), 8.862 (1H, s, CH_3_-N = CH-Ar), 8.438–8.418 (1H, d, *J* = 8, Ar-H), 8.355–8.307 (2H, dd, *J* = 8.8, 3.6, Ar-H), 8.114–8.094 (1H, d, *J* = 8.0, Ar-H), 8.041–8.018 (1H, d, *J* = 8.0, Ar-H), 7.891–7.869 (1H, d, *J* = 8.8, Ar-H), 7.731–7.694 (1H, t, *J* = 7.2, Ar-H), 7.622–7.585 (1H, t, *J* = 8.0, Ar-H), 7.483–7.461 (1H, t, *J* = 8.8, Ar-H), 7.217–7.198 (2H, m, Ar-H), 5.192 (2H, s, -N-CH_2_), 4.211–4.158 (2H, q, *J* = 7.2, -O-CH_2_), 2.505 (3H, s, -CH_3_), 1.249–1.214 (3H, t, *J* = 7.2, -CH_3_); ^**13**^**C NMR** (100 MHz, DMSO-*d*_*6*_): δ ppm:168.602, 153.88, 152.217, 148.131, 145.138, 141.430, 137.031, 130.101, 128.833, 128.178, 127.482, 127.271, 125.388, 124.674, 123.720, 122.755, 122.242, 121.368, 120.985, 112.485, 109.656, 109.539, 108.235, 61.201, 44.431, 14.026, 9.961. **ESIMS** found: *m*/*z* 452.6 [M-H]^-^, 454.6 [M+H]^+^, 476.5 [M+Na]^+^_;_ **R**_***f***_ = 0.50 (hexane: ethyl acetate = 1:2).

Human epithelioid cervical carcinoma HeLa cells, human breast adenocarcinoma MCF7 and MDA-MB-231 cells, human hepatocellular carcinoma HepG2 and Hep3B cells, human colon adenocarcinoma HCT-116 and HT-29, human epidermoid carcinoma A431 cells, human glioblastoma A-172, SnB-75, and U-373MG cells, human prostate carcinoma PC-3 cells, human leukemia K562 and HL-60 cells, human lung carcinoma A549 and NCI-H460 cells, and human osteosarcoma U-2OS cells were purchased from ATCC. Human gastric carcinoma SNU-484 and SNU-601 cells were obtained from Korean Cell Line Bank. The detail characteristics of parental MCF7 and K562 cell lines and the multidrug-resistant, P-glycoprotein overexpressing MCF7/ADR and K562/ADR cell lines were obtained from Bio-Evaluation Center at KRIBB [15, 16].

The male FVB/N mice were purchased from The Jackson Laboratory. All animal care and experimental protocols used in this study were approved by the Institutional Animal Care and Use Committee of KRIBB (permit number: KRIBB-AEC-16081). According to KRIBB guidelines for the care and use of laboratory animals, the mice were housed individually in the standard cages, and maintained at 22 ± 2°C in a room with a 12-hour light/dark cycle. Fresh food and water were provided at all times. All procedures were performed under anesthesia by inhalation of Isoflurane, and all efforts were made to minimize suffering.

### Chemical screening methods

All compounds from a small-molecule library obtained from Korea Chemicals Bank were evaluated for their antiproliferative activity on various cancer cell lines. The MTT assay was used to determine the cytotoxic effect of compounds and each IC_50_ value was assessed by log-dose-response curves. Also, the EZ-CyTox cell viability assay (Daeil Lab. Service, Korea) was performed according to the manufacturer’s instructions and the absorbance was measured at 450 nm using VersaMax^™^ (Molecular Devices LLC, USA). Each IC_50_ value was calculated using a nonlinear regression analysis using GraphPad Prism 6.0 program.

Considering the functions of microtubule in the maintenance of cellular morphology, further analyses that involved the disruption of cellular morphology was performed by microscopic examination and immunocytochemistry. The detailed methods including flow cytometric analysis are described in the following sections.

### Flow cytometric analysis

Following compound treatment, cells were harvested and stained with propidium iodide (PI) according to the instruction of Cycletest Plus DNA Reagent Kit (BD Biosciences) or with anti-Annexin V-FITC (BD Biosciences) for 30 min to determine the percentage of cells with phosphatidylserine externalization. Flow cytometric analysis was performed using a FACSCalibur instrument (BD Biosciences).

### Immunoblot and immunofluorescence staining

Cells were lysed with cold RIPA buffer and whole cell lysates were subjected to SDS-PAGE as previously reported [17]. To perform immunofluorescence staining, the cells were fixed and incubated with Alexa 488-conjugated α-Tubulin antibody. DNA was stained with Hoechst 33342 in PBS. Images were analyzed on a fluorescence microscope (Nikon Instruments Inc.).

### Tubulin polymerization assay

The assay was performed according to the manufacturer’s instructions (Cytoskeleton, Inc., USA). In brief, tubulin proteins (>97% pure) were suspended in G-PEM buffer (80 mM PIPES (pH 6.9), 2 mM MgCl_2_, 0.5 mM EDTA, and 1.0 mM GTP) to a final concentration of 4.0 mg/mL. The tubulin solution was then incubated with G-PEM buffer alone (control), and STK899704 (10 μM, final concentration) at 37°C. Paclitaxel and vinblastine at final concentration of 5 μM were also used as positive enhancer and inhibitor controls, respectively. The polymerization of tubulin was measured by continuous monitoring of the turbidity change at 340 nm (VersaMax^™^).

### Computer modeling study

To examine the binding mode of STK899704 with respect to impairing the activity of tubulin, we conducted docking simulations in the active site. Three dimensional atomic coordinates were extracted from the X-ray crystal structure of tubulin (PDB code: 1SA0) as the receptor model [18]. Gasteiger-Marsili atomic charges were determined for all the protein and ligand atoms to calculate the electrostatic interactions between tubulin and STK899704 [19]. Docking simulations to address the binding mode of STK899704 were then carried out with the modified version of AutoDock program whose outperformance had been well appreciated for various target proteins [20–23]. Of the twenty binding conformations of STK899704 generated with docking simulations, those differing by less than 1.5 Å in positional root-mean-square deviation were clustered together. The lowest-energy binding configuration in the top-ranked cluster was finally selected for further analysis.

### Tubulin competitive binding SPA assay

The competitive binding SPA assay was performed as the manufacturer’s instructions (Cytoskeleton, Inc.) using biotin-labeled tubulin, streptavidin-coated PVT SPA beads (Perkin Elmer), and Colchicine [ring C, methoxy-^3^H] (1 mCi/mL, specific activity 85 Ci/mmol) (American Radiolabeled Chemicals, Inc.). Briefly, lyophilized biotin-labeled tubulin was incubated with streptavidin-coated PVT SPA beads for 30 min at 4°C, and the premix beads were then incubated with [^3^H]colchicine (30 nM) and various concentrations of unlabeled colchicine, STK899704, or vinblastine (0.1, 0.3, 1.0, 3.0, 10.0, 30.0, and 100 μM final concentration) for additional 45 min at room temperature. The scintillations were then measured using 1450 MicroBeta TriLux (Perkin Elmer).

### Skin carcinogenesis *in vivo*

A two-stages carcinogenesis was performed as previously described [24, 25]. The dorsal skin area of the 6–7 week old mice was shaved 2 days before start of the experiment. Tumorigenesis was initiated by a single topical treatment with 100 μg of DMBA in 0.2 ml of acetone over a period of 1 week. Tumor promotion was then induced by treatment with 5 μg of TPA in 0.2 ml of acetone twice weekly. In order to measure the number and volume of skin tumors, mice were weighed and photographed every week starting from when first measurable tumors (1 mm^3^) appeared. Tumor volume was calculated using the following formula: tumor volume 4π/3(*l*/2)(*w*/2)(*h*/2), where *l* is the length, *w* is the width, and *h* is the height [19]. At the end of the experiment, the mice were euthanized with CO_2_, and both tumors and skin were collected for histological and biochemical studies.

Within 30 minutes following the above dose of TPA application, STK899704 or 5-FU at 500 nM in 0.2 ml of acetone was treated twice a week over 15 weeks. The mice were divided into four groups and each group consisted of more than 20 mice. Two groups of mice were treated with acetone (vehicle) or TPA only, which served as negative or positive controls, respectively.

### Test for adverse effects of STK899704 treatment

To test adverse effects of STK899704 treatment, the compound was applied onto the shaved area of dorsal skin in healthy group of mice twice weekly, 20 times in total. The effects were compared to control group treated with acetone only, and each group was consisted of more than 10 mice.

Collected samples of skin were fixed at 4°C in 4% paraformaldehyde/PBS or 10% formalin solution and then sectioned for histological analyses [15]. The sections (10 μm thickness) were stained with hematoxylin/eosin and observed using research system microscope BX51 (Olympus).

## Results

### Antiproliferative effect of STK899704 on various cancer cells and multidrug-resistant cell lines

**Fig 1:**
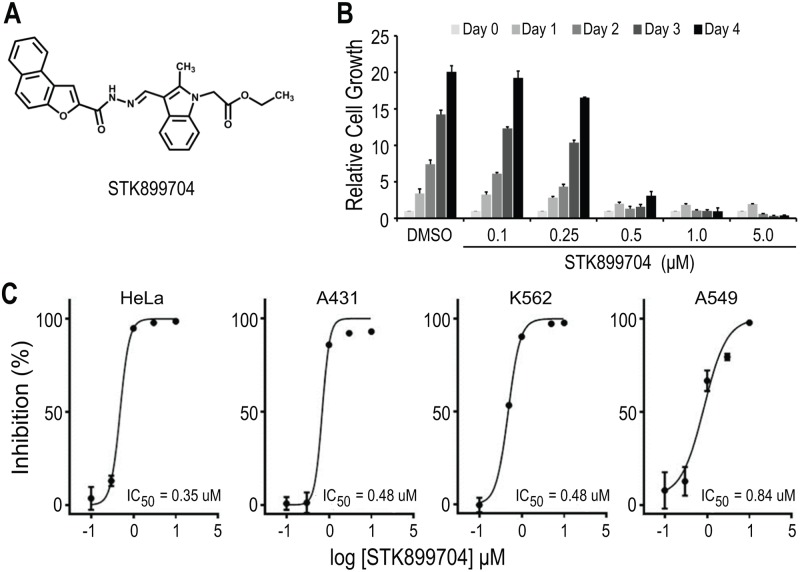
STK899704 suppressed the growth of a variety of human cancer cell lines. (A) Chemical structure of STK899704. (B) Antiproliferative effect of STK899704 on HeLa cells. Cells were seeded at 2 x 10^3^ cells in 96-well plate and treated with various concentrations of STK899704 for 4 days. Cell growth was determined by MTT assay. (C) Inhibitory effects of STK899704 on the growth of various cancer cell lines. Data were fitted with dose-response curve by using Graphpad Prism software.

In the course of screening for antiproliferative agents from a small-molecule library, we found that ethyl(2-methyl-3-((E)-((naphtha(2,1-b)furan-2-ylcarbonyl)hydrazono)methyl)-1H-indole-1-yl)acetate or STK899704 (Fig 1A) displayed a potent antiproliferative effect against HeLa cervical cancer cells as shown in Fig 1B. STK899704 suppressed the growth of HeLa cells in a dose-dependent manner with an IC_50_ of 350 nM (Fig 1C). Furthermore, STK899704 inhibited the growth of a variety of human cancer cell lines including skin, bone, breast, colon, prostate, lung, stomach, brain, and liver cancers and leukemia with IC_50_ values ranging from 0.35 to 1.54 μM (Fig 1C and S1 Table).

One major mechanism of acquired resistance to anticancer drug is mediated by overexpression of drug-efflux protein P-glycoprotein [8]. The antiproliferative activity of STK899704 was compared with doxorubicin and Taxol in K562, MCF7, and their respective P-glycoprotein overexpressing multidrug-resistance (MDR) cell lines, K562/ADR and MCF7/ADR [15, 16]. The MDR cells were resistant to doxorubicin, Taxol, vinblastine, and colchicine with resistance factors (ratio of IC_50_ of resistance cell line relative to its parental cell line) ranging from 7.6 to 582 fold (Table 1), whereas STK899704 exhibited a potent cytotoxic effect against these MDR cell lines as judged by resistance factors of 0.12 and 0.16 for K562/ADR and MCF7/ADR, respectively.

**Table 1:** Effect of STK899704, doxorubicin, Taxol, vinblastine, and colchicine on multidrug-resistant cell lines.

Cell line	IC_50_ (μM)				
	STK899704	Doxorubicin	Taxol	Vinblastine	Colchicine
K562	0.360	0.021	0.002	0.026	0.010
K562/ADR	0.044 (0.12)	2.925 (139.3)	1.164 (582)	0.442 (17)	0.451 (45.1)
MCF7	1.610	0.063	0.005	0.024	0.028
MCF7/ADR	0.262 (0.16)	3.767 (59.8)	1.089 (217.8)	0.183 (7.6)	0.454 (16.2)

K562, MCF7 and their MDR cell lines were cultured in microtiter plates (1~2 × 10^3^ cells/well) and incubated with different concentrations of STK899704 for 4 days. The MTT assay was performed to determine the cytotoxic effect of STK899704 and IC_50_ was assessed by log-dose-response curve. The resistant factor (in parenthesis) was calculated by the ratio of the IC_50_ of the resistant to that of the parental cells. All values represent mean ± SDs from triplicate experiments.

### STK899704 induced mitotic arrest

**Fig 2:**
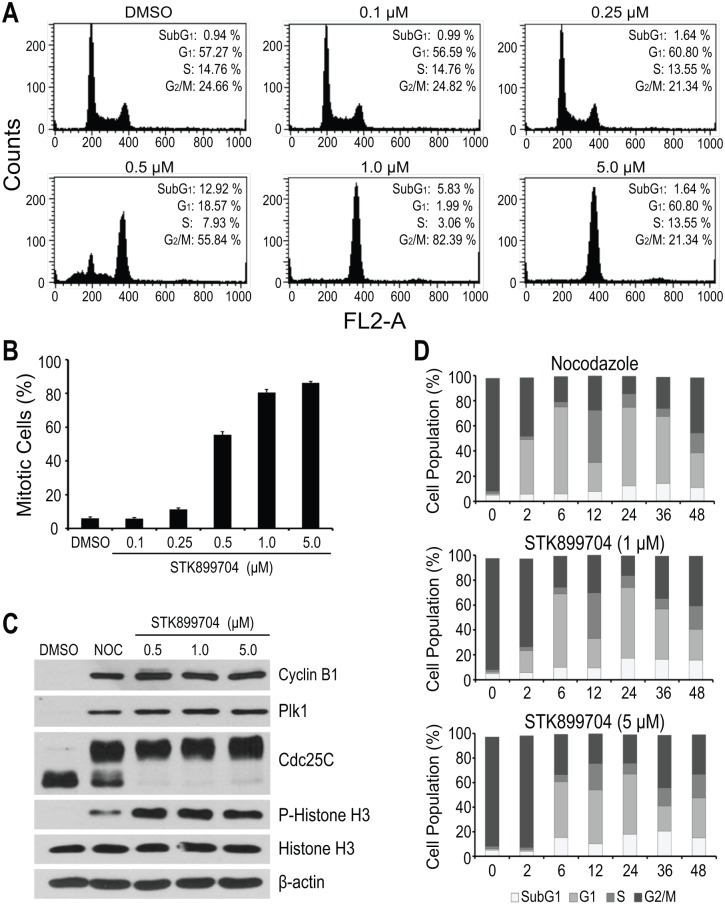
Antimitotic effect of STK899704. (A) Flow cytometric analysis for cell cycle distribution. HeLa cells were treated with the indicated concentrations of STK899704 for 24 h. Treated cells were then stained with propidium iodide (PI) and processed for cell cycle analysis. Data are representative of three independent experiments. (B) Mitotic index. HeLa cells were treated with the indicated concentrations of STK899704 for 17 h. Cells were then stained with Hoechst 33342 and mitotic cells were counted. At least 100 cells were counted from the different regions. (C) Cell cycle related protein expression. HeLa cells were treated with DMSO control, 200 ng/ml nocodazole (Noc), or indicated concentrations of STK899704 for 17 h. Treated cells were lysed and subjected to immunoblot analysis with antibodies against cyclinB1, Plk1, Cdc25C, histone H3, and phospho-histone H3 (S10). β-actin was used as a loading control. (D) Reversible effect of STK899704. HeLa cells were treated with nocodazole (200 ng/ml) or STK899704 (1 or 5 μM) for 17 h. Cells were washed twice and released into fresh DMEM without nocodazole or STK899704. Cells were then stained with PI at the indicated time and processed for cell cycle analysis. Each Bar indicates mean ± SD from three independent experiments.

The treatment of HeLa cells with STK899704 resulted in a dose-dependent accumulation of G_2_/M phase cells with 4N DNA content and, concomitant decrease in G_1_ and S phase cells (Fig 2A). The increased G_2_/M phase cells were accompanied by a marked increase in rounded, mitotic-like cell morphology, suggesting that STK899704 might induce mitotic arrest. To address this, we assessed the percentage of mitotic cells by quantification of the number of cells with highly condensed chromosomes, characteristic of particular mitotic chromosomes [26] and found that STK899704 treatment resulted in a dose-dependent increase in mitotic indices (Fig 2B). Hence, these results indicate that STK899704 causes cell cycle arrest at M phase.

In addition, we evaluated the effect of STK899704 on cell cycle related proteins, including Cdc25C, histone H3, cyclin B1, and polo-like kinase (Plk1), in comparison with nocodazole, a tubulin depolymerizing agent and well-known mitotic blocker [6]. During mitosis, Cdc25C activity is hyperphosphorylated causing its slower migration on SDS-PAGE [27, 28], whereas the phosphorylation of histone H3 at S10 is required for proper chromosome condensation and segregation [29, 30]. Both Cyclin B1 and Plk1 expression oscillate throughout the cell cycle. Their levels are minimal in G_1_ phase, begin to accumulate in S phase, and reach the maximum at G_2_/M boundary [31–34]. As expected, the treatment of HeLa cells with nocodazole, as well as STK899704, resulted in elevated Cdc25C and histone H3 phosphorylation levels and accumulated cyclin B1 and Plk1 levels (Fig 2C), confirming that STK899704 induces mitotic arrest.

To examine whether the antimitotic activity of STK899704 is reversible, HeLa cells were treated with STK899704 (1 and 5 μM) for 18 h. Nocodazole, a reversible inhibitor of microtubule assembly [35], was included as control. As shown in Fig 2D, HeLa cells were efficiently arrested at G_2_/M phase (>80%) upon treatment with nocodazole and STK899704 overnight. After the removal of nocodazole and STK899704, the G_2_/M-arrested HeLa cells were able to re-enter the cell cycle with a minimal delayed in cells treated with a high concentration of STK899704 (5.0 μM) (Fig 2D). These results indicated that STK899704, like nocodazole, acts in a reversible manner.

### STK899704 interfered with tubulin polymerization and mitotic spindle organization

**Fig 3:**
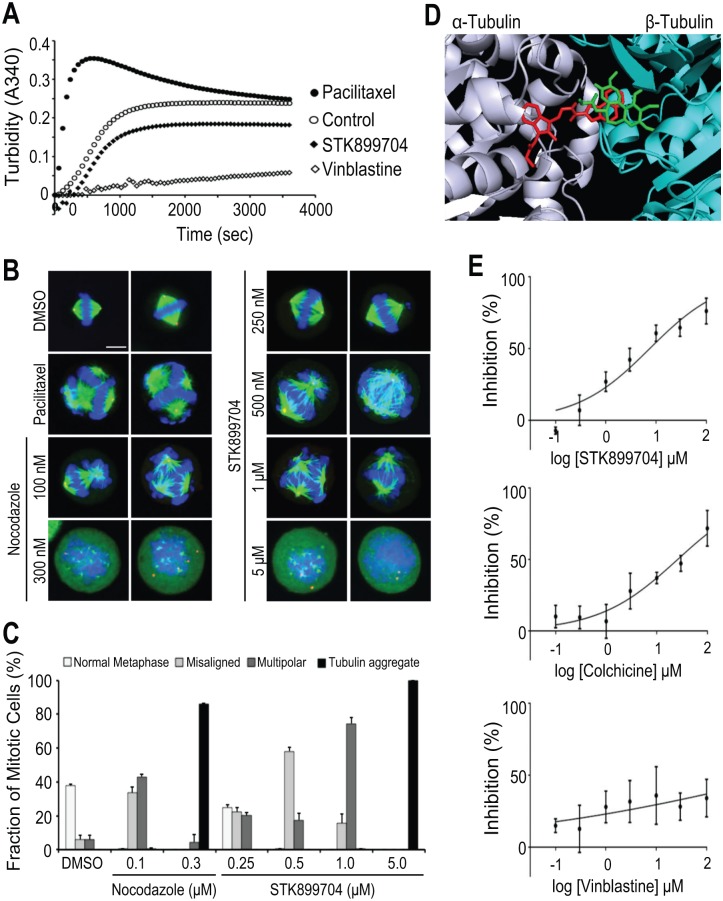
STK899704 inhibited tubulin polymerization and mitotic spindle organization. (A) Tubulin polymerization assay. The effect of STK899704 (5 μM) on polymerization of purified tubulin *in vitro* was examined in a GTP-containing buffer. DMSO was used as a negative control. Tubulin-targeting agents Taxol (5 μM) and vinblastine (5 μM) were also used as controls for tubulin-stabilizing and tubulin-destabilizing agents, respectively. Assembly of tubulin into microtubules was determined by the degree of turbidity at 340 nm. (B) Immunofluorescence staining of microtubules in HeLa cells. Cells were treated with DMSO, Taxol (100 nM), nocodazole (200 ng/ml), or indicated concentrations of STK899704 for 17 h. Cells were then fixed and stained with Alexa Fluor 488-conjugated anti-tubulin antibody and Hoechst 33342 to visualize α-tubulin and DNA, respectively. Scale bar, 10 μm. (C) Fraction of mitotic cells. At least 100 cells from (B) were counted from the different regions. Percentages of normal metaphase, misaligned, multipolar, and tubulin aggregate phenotypes were shown. (D) Proposed binding model of STK899704 on tubulin. The αβ-tubulin heterodimer from PDB entry 1SA0 is shown as ribbon (gray, α-tubulin; cyan, β-tubulin). STK899704 is presented in red stick while colchicine is shown in green. (E) Effect of STK899704 on tubulin binding. Tubulin binding was tested with a SPA-based competition assay. Error bars represent mean ± SDs from three independent experiments.

Given the fact that microtubules are the major structural component of a mitotic spindle, whose function is critical for chromosome segregation during mitosis [2], and that a large number of antimitotic agents interact with tubulin and thereby alter its polymerization and dynamics [5], we examined the effect of STK899704 on tubulin polymerization *in vitro* in comparison with known microtubule binding agents by tracking the change in turbidity over time. In the absence of treatment, tubulin heterodimers self-assembled to form linear tubulin polymers in a time-dependent manner as shown in Fig 3A. Treatment with the microtubule-stabilizing agent paclitaxel resulted in enhanced tubulin polymerization, whereas the microtubule-depolymerizing agent vinblastine, as well as STK899704, interfered with tubulin polymerization (Fig 3A).

To further determine whether STK899704 could affect mitotic spindle organization in cells, HeLa cells were treated with DMSO control or test compounds, followed by formaldehyde fixation and subsequent staining to visualize α–tubulin, γ-tubulin, and chromosomes. As shown in Fig 3B, Taxol, a microtubule stabilizing agent, significantly induced multipolar spindles with highly condensed chromosomes resulting from enhanced tubulin polymerization. Abnormal spindle morphology was also clearly observed in cells treated with lower concentrations of nocodazole (0.1 μM) and STK899704 (0.25–1.0 μM) (Fig 3B and 3C). In some instances, spindle appeared normal but chromosomes were not aligned at the metaphase plate. In others, multipolar spindles were observed. This abnormal spindle morphology suggests that nocodazole and STK899704 at low concentration interfere with spindle microtubule dynamics. However, cells treated with higher concentrations of nocodazole (0.3 μM) or STK899704 (5.0 μM) exhibited condensed chromosome with aggregated tubulin and disrupted microtubules. Taken together, these results suggest that STK899704, like nocodazole, is a microtubule-depolymerizing agent.

We next search for potential binding site of STK899704 on tubulin. Using computer modeling and X-ray structure PDB code 1SA0 [18], we determined the proposed binding site of STK899704 on tubulin (Fig 3D, red stick) is located between the α- and β-subunit of tubulin at the colchicine binding site (Fig 3D, green stick). Comparison of their proposed binding modes showed that colchicine binds to the β-tubulin subunit whereas the STK899704 ligand spans across the intra dimer interface suggesting that the binding site of STK899704 somehow overlaps with colchicine. We confirmed that STK899704 interacts directly with this binding site using a tubulin competitive binding SPA assay. In addition, we showed that STK899704 competitively inhibited [^3^H]colchicine binding to biotinylated tubulin, similar to unlabeled colchicine, whereas vinblastine did not significantly influence the binding of [^3^H]colchicine (Fig 3E). Thus, these data indicate that STK899704 is a new class of tubulin inhibitor; and its antiproliferative activity is due to its binding to tubulin at the colchicine-binding site.

### STK899704 induced cell death following prolonged mitotic arrest

**Fig 4:**
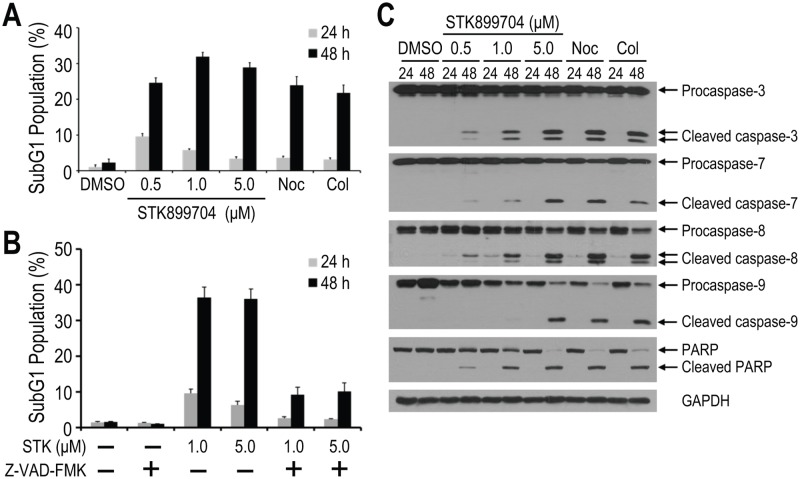
STK899704 triggered programmed cell death. (A) Effect of STK899704 on DNA fragmentation. HeLa cells were treated with 200 ng/ml nocodazole (Noc), 100 nM colchicine (Col), or indicated concentrations of STK899704. Treated cells were then stained with PI and processed for cell cycle analysis at 24 and 48 h. (B) Antagonistic effect of Z-VAD-FMK on STK899704-induced cell death. HeLa cells were treated with DMSO or STK899704 (STK, 1 or 5 μM) in the presence or absence of Z-VAD-FMK (50 μM). Cells were then stained with PI and processed for cell cycle analysis at 24 and 48 h. (C) Effect of STK899704 on the levels of activated caspases. HeLa cells were treated as in (A) and then subjected to immunoblot analysis with antibodies against caspase-3, caspase-7, caspase-8, caspase-9, and PARP. GAPDH was used as a loading control. Each bar indicates mean ± SD of subG_1_ population from three independent experiments.

We next examined whether STK899704 would eventually induce cell death after prolonged mitotic arrest by determining the subG_1_ populations of HeLa cells. Similar to the effect of colchicine and nocodazole, treatment with STK899704 resulted in a marked increase in the subG_1_ population at 48 h (Fig 4A). Since caspases are the key mediators of apoptosis [36], Z-VAD-FMK, a cell-permeable and irreversible pancaspase inhibitor, was used to examine whether caspases are involved in the increased subG_1_ population after STK899704 treatment. As shown in Fig 4B, co-treatment with Z-VAD-FMK prevented the accumulation of the subG_1_ population compared with STK899704 treatment alone. These results suggest that STK899704 treatment induces apoptosis in a time-dependent manner, associated with the increase in the subG_1_ population.

In addition, the effects of STK899704, colchicine, and nocodazole on the cleavage of initiator caspases were analyzed. Caspase-3 and caspase-7 are the effector caspases that are activated by initiator caspases such as caspase-8 and caspase-9. As shown in Fig 4C, the cleaved caspases were clearly detectable at 48 h in a concentration-dependent manner. The level of PARP cleavage was also examined since activation of effector caspases such as caspase-3 leads to downstream cleavage of various substrates including poly (ADP-ribose) polymerase (PARP) during apoptosis [36, 37]. Consistent with active caspase levels, PARP cleavage was also prominent at 48 h after treatment with STK899704, colchicine, and nocodazole (Fig 4C). Taken together, these results indicate that STK899704 induces prolonged mitotic arrest and consequently leads to apoptosis.

### STK899704 demonstrated prominent antitumor activity *in vivo*

Due to the physicochemical properties of STK899704, which is very lipophilic (XlogP3 = 5.7, pubchem), and the efficient antiproliferative activity shown earlier, we concluded that STK899704 might be an effective local antitumor agent when directly applied to the skin. Therefore, we assessed the antitumor effect of STK899704 in a skin carcinogenesis model *in vivo* and used 5-fluorouracil (5-FU), a widely used therapeutics for skin cancer, as a comparison treatment in this study.

**Fig 5:**
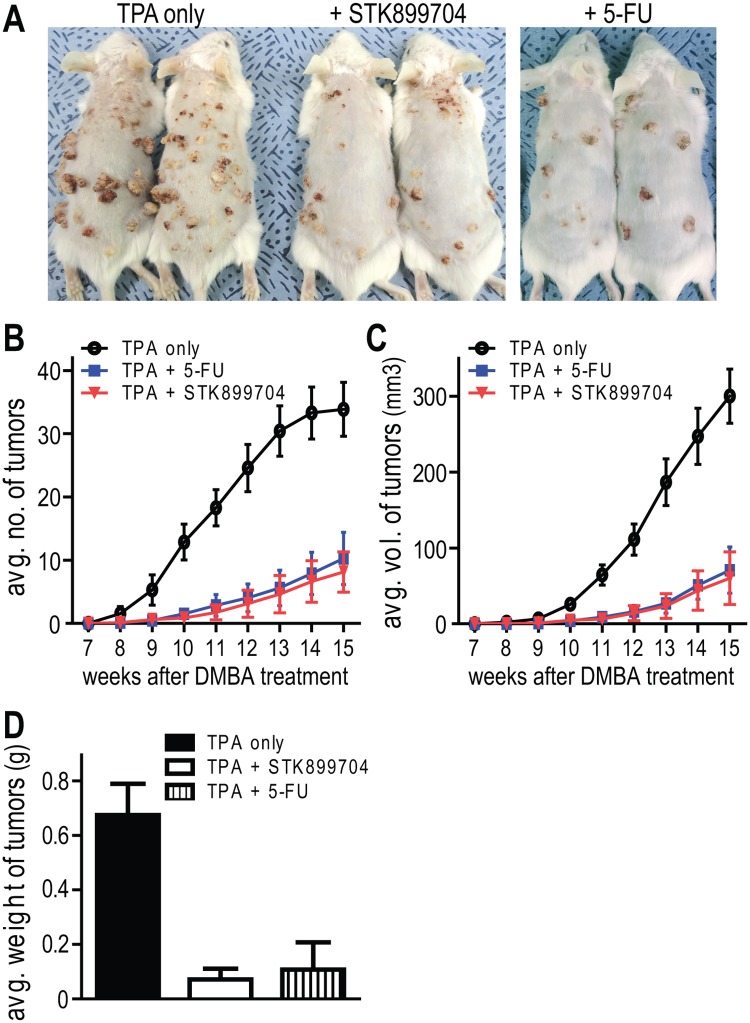
The antitumor activity of STK899704 *in vivo* carcinogenesis model. After tumor initiation by DMBA treatment, mice were further subjected to TPA challenge for tumor promotion twice a week. Either STK899704 or 5-fluorouracil (5-FU) was applied topically 30 minutes after TPA treatment for 15 weeks. (A) The tumor development and growth were assessed during STK899704 or 5-FU treatment and compared to TPA-treated control group at 15th week. Average number (B) and volume (C) of skin tumors per group were weekly recorded and calculated during treatment. (D) Average weight of tumors was measured in each group of mice at the end of the experiment. Error bars show mean ± SDs for each group of mice.

After tumor initiation by DMBA treatment, mice were treated with STK899704 or 5-FU was co-treated with TPA to group of mice twice a week for 15 weeks. As shown in Fig 5A, TPA treatment alone resulted in the formation of many tumors on the back of the mice. In contrast, both number and size of tumors were significantly decreased by STK899704 or 5-FU treatment, exhibiting an antitumor promotion effect of STK899704. For statistical analyses, changes in total number and volume of tumors were weekly monitored during treatment (Fig 5B and 5C). Surprisingly, STK899704 treatment exhibited approximately 76% reduction in the average number of DMBA/TPA-induced skin tumors, while 5-FU treatments were found to decrease skin tumors by about 70% compared to the TPA treatment alone at 15 weeks (Fig 5B). Similarly, STK899704 treatment reduced the tumor volume by approximately 80%, while 5-FU treatments decreased the tumor volume by about 76% at 15 weeks (Fig 5C). At 15 weeks, skin tumors from each group of mice were isolated, and their weight was measured as shown in the Fig 5D. The mean weight of the skin tumors was obviously decreased 9.4-fold in the STK899704-treated group. These results indicate that STK899704 exhibits potent both chemopreventive and antitumor effects, and the efficacy of STK899704 showed beyond that of 5-FU.

The effects of STK899704 treatment on normal skin were also evaluated in healthy group of mice. Visual inspection and histological examination of the skin sections revealed that there were no abnormal symptoms such as skin inflammation and hyperplasia (S2 Fig).

## Discussion

In our effort to discover novel antiproliferative agents, we identified STK899704 as a novel tubulin inhibitor. STK899704 has an acylhydrazone moiety known as a useful scaffold for drug development, especially for anticancer therapy due to its antimitotic activity [38–42]. Further analyses showed that STK899704 strongly induces the accumulation of mitotic cells in a concentration-dependent and reversible manner (Fig 2A, 2B and 2D). These results suggest that STK899704 induces cell cycle arrest in mitosis. In addition, the antimitotic effect of STK899704 was confirmed by increased phosphorylation of Cdc25C and accumulation of cyclin B1 and Plk1 (Fig 2C), which are associated with mitosis [18, 27, 28, 31–37]. Moreover, prolonged mitotic arrest leads to a time-dependent increase in subG_1_ population (Fig 4A) and activation of caspases (Fig 4C). The effector caspases are implicated in the late, irreversible phase of the apoptotic pathway and carry out the proteolytic degradation of a broad range of cellular targets, such as PARP which plays an important role in diverse cellular processes, including DNA damage response [36, 37]. Therefore, increased DNA fragmentation and altered levels of active caspases suggest that prolonged mitotic arrest induced by STK899704 eventually trigger programmed cell death.

We also found that the antimitotic activity of STK899704 is ascribed to its ability to prevent tubulin polymerization *in vitro* and disrupt microtubules in cells (Fig 3A and 3B), indicating that STK899704 is a microtubule-destabilizing agent. Computer modeling studies reveal that STK899704 possibly binds to the colchicine-binding site (Fig 3D). The colchicine ligand lies in β-tubulin subunit whereas STK899704 ligand traverses across the interface between αβ-tubulin heterodimer. Additionally, the results from Figs 2D and 3D suggest that STK899704 does not covalently bind to tubulin, allowing for the full reversibility of its intracellular activity.

One of the major limitations of anticancer drugs in clinical use is drug resistance, acquired or intrinsic, especially resistance due to the expression of the drug-efflux protein P-gp [8]. Unlike Taxol and the Topoisomeras II inhibitor doxorubicin, STK899704 exhibits equal or better potency in both parental and multidrug-resistant cells overexpressing P-glycoprotein. Therefore, STK899704 may overcome the resistance mediated by the multidrug resistance protein P-gp.

In this study, the efficacy of STK899704 was investigated using a DMBA/TPA-induced skin carcinogenesis model (Fig 5). Skin cancer is a generic term for malignant tumor occurring on the skin and its incidence rate has been generally on the rise [43, 44]. The overall prevalence of skin cancer has been reported to be higher in Caucasians and becomes higher with age, in part due to the accumulative exposure to UV light [45–47]. Thus, skin cancer is emerging as a global issue in terms of disease prevention ranging from Europe and the United States whose major races are Caucasians, to Asia where the population age is steadily rising. Therefore, development of a therapy with convenient applicability and low side effect would be in high demand. Here we reported that topically applied STK899704 exhibited efficient antitumor effect. In response to STK899704 treatment, approximately 80% of the total number of skin tumors dramatically decreased (Fig 5B). Compared to the TPA-treated control group, tumors size was also reduced to approximately 80% by STK899704 treatments (Fig 5C). Thus, the prominent antitumor activity of STK899704 has been proved *in vivo* animal model.

Except surgical removal of the lesion, 5-FU and Imiquimod are the most widely used topical therapeutics for skin cancer [48, 49]. However, 5-FU is occasionally accompanied by many side effects including local pain, itchiness, burning, stinging, and dermatitis. Imiquimod acts as immune response modifier that can stimulate the immune system to primarily produce interferon-α (INF-α) leading to nonspecific inflammation or dermatitis. Since the antitumor effect of STK899704 was surprisingly comparable to 5-FU (Fig 5) and did not induce abnormal symptoms or alteration in skin conditions (S2 Fig), STK899704 is a promising alternative to 5-FU or Imiquimod for treatment of skin cancer.

Taken together, our data indicate that the antiproliferative effect of STK899704 is ascribed to its microtubule destabilizing activity, resulting in mitotic arrest and apoptosis. In addition, the antiproliferative activity of STK899704 seems to be effective regardless of any multidrug-resistance phenotype. Our study suggests that STK899704 is a promising candidate for further development as a chemotherapeutic agent, particularly for the treatment of skin cancer as well as MDR cancer.

## Supporting information

S1 Fig Reaction scheme for the synthesis of STK899704. Synthesis of (E)-ethyl 2-(2-methyl-3-((2-(naphtho[2,1-b]furan-2-carbonyl)hydrazono)methyl)-1H-indol-1-yl)acetate (STK899704) is outlined in **Scheme 1**. The preparation of the title compound STK899704 was started from 2-Hydroxy-1-naphthaldehyde (**1)** and ethyl bromoacetate to obtaine Naphtho[2,1-b]furan-2-carboxylic acid (**2a**). This reaction required anhydrous K_2_CO_3_ as base and dimethylformamide (DMF) as solvent, both condensation as well as cyclization occurred in single step in 90% yield. The structure of the product **2a** was determined by ^1^H NMR and ESIMS analyses. The 7 aromatic protons characteristic signals appeared between δ8.269–7.460 ppm in ^1^H NMR and 13 carbon signals appeared between δ 161.293–105.238 ppm in ^13^C NMR spectra, also its mass spectrum revealed a molecular ion peak at *m*/*z* 211.4[M-H]^-^corresponding to the molecular formula C_13_H_8_O_3_ confirms the structure of **2a**. In the second step of reacton, Naphtho[2,1-b]furan-2-carboxylic acid (**2a**) converted to the corresponding ester ethyl naphtho[2,1-b]furan-2-carboxylate (**2b**) in 60% yield, by esterification reaction using ethanol and SOCl_2_. The carboxylate **2b** was confirmed by the presence of new peaks quartet—CH_2_ at δ 4.424–4.370 ppm (*J* = 7.2 Hz) and triplet—CH_3_ at δ 1.388–1.353 ppm (*J* = 7.2 Hz) along with 7 aromatic protons in ^1^H NMR spectrum and also by molecular ion peak at *m*/*z* 241.4 [M+H]^+^, 263.4 [M+Na]^+^ appeared in ESIMS spectrum corresponding to the molecular formula C_15_H_12_O_3_.Thus compound **2b** was reacted with hydrazine hydrate in third step of reaction to obtain an intermediate compound naphtho[2,1-*b*]furan-2-carbohydrazide **3** in 72% yield. ^1^H NMR spectrum of compound **3** exhibited no peak corresponds to ester instead it shows signals at δ 10.049 ppm for amide O = C-NH and δ 4.597 ppm for -NH_2_ (D_2_O exchangeable) of hydrazide respectively. The structure was further confirmed by recording its mass spectra, by its molecular ion peak at *m*/*z* 225.4 [M-H]^-^, 227.4 [M+H]^+^, 249.4 [M+Na]^+^corresponds to molecular formula C_13_H_10_N_2_O_2_. Vilsmeier-Haack formylation of 2-methyl indole (**4**) gave 2-methyl-1H-indole-3-carbaldehyde (**5**) in 87% yield in fourth step. Reaction of phosphorous oxychloride with DMF at low temperature led to the formation of an electrophile and then was slowly added 2-methyl indole. The structure of the product **5** was confirmed by the appearance of new peak at δ 10.058 ppm for—CHO instead of one aromatic proton in ^1^H NMR spectrum. ESIMS analysis data indicates the molecular ion peak at *m*/*z* 158.4 [M-H]^-^, 160.4 [M+H]^+^, 182.3 [M+Na]^+^ which is corresponding to molecular formula C_10_H_9_NO. The intermediate compound ethyl 2-(3-formyl-2-methyl-1H-indol-1-yl)acetate (**6**) was obtained in 86% yield in step 5, by *N*-substitution of 2-methyl-1H-indole-3-carbaldehyde (**5**) by ethyl bromoacetate using sodium hydride as a base in THF solvent and it was confirmed by the absence of indole—NH signal at δ 11.962 ppm instead appeared new signals at δ 4.208–4.155 ppm, quartet for—CH_2_ and δ 1.241–1.205 ppm triplet for—CH_3_ in ^1^H NMR spectrum and also by molecular ion peak at *m*/*z* 244.4 [M-H]^-^, 246.4 [M+H]^+^, 268.4 [M+Na]^+^ appeared in ESIMS spectrum corresponding to the molecular formula C_14_H_15_NO_3_. Finally the title compound (E)-ethyl 2-(2-methyl-3-((2-(naphtho[2,1-b]furan-2-carbonyl)hydrazono)methyl)-1H-indol-1-yl)acetate (STK899704) was obtained in 75% yield, by condensation of hydrazide **3** with aldehyde **6** in presence of catalytic amount of acetic acid in absolute ethanol. The structure of newly synthesized molecule STK899704 was confirmed by ^1^H NMR, ^13^C NMR and mass analyses. ^1^H NMR of compound STK899704 exhibits singlet at δ 11.838 ppm for amide proton O = C-NH-, singlet at δ 8.862 ppm for azomethine proton—CH = N-, 11 aromatic protons appeared between δ 8.438–7.198 ppm, δ 5.192 ppm for -N-CH_2_, δ 4.211–4.158 ppm for—O-CH_2_-,δ 1.249–1.214 ppm for—CH_3_, and δ 2.505 ppm singlet appeared for—CH_3_ attached to aromatic ring.^13^C NMR spectrum of compound STK899704 exhibits total 27 carbon signals. The structure was further confirmed by recording its mass spectrum, by its molecular ion peak at *m*/*z m*/*z* 452.6 [M-H]^-^, 454.6 [M+H]^+^, 476.5 [M+Na]^+^ corresponds to molecular formula C_27_H_23_N_3_O_4_.
(TIF)

S2 Fig The effects of STK899704 treatment on normal skin. To examine additional effects of STK899704 treatment on normal skin, the compound or acetone was applied onto the dorsal skin of healthy mice twice weekly for 10 weeks. (A) The picture taken after the last treatment. (B) Hematoxylin/eosin stained sections from skin samples exhibit no skin abnormalities caused by STK899704 treatment.
(TIF)

S1 Table Antiproliferative activity of STK899704 on various cancer cell lines. The various cancer cell lines were cultured in microtiter plates (1–2 x 10^3^ cells/well) and incubated with different concentration of STK899704 for 4 days. The MTT assay was used to determine the cytotoxic effect of STK899704 and IC_50_s were assessed by log-dose-response curves. Data are the average of triplicate assays.
(PDF)
