# Supplementary figures and images for: Anticancer activity of a novel small molecule tubulin inhibitor STK899704
Source: PLoS One. 2017 Mar 15;12(3):e0173311. doi: 10.1371/journal.pone.0173311 (PMC5351965; doi:10.1371/journal.pone.0173311)

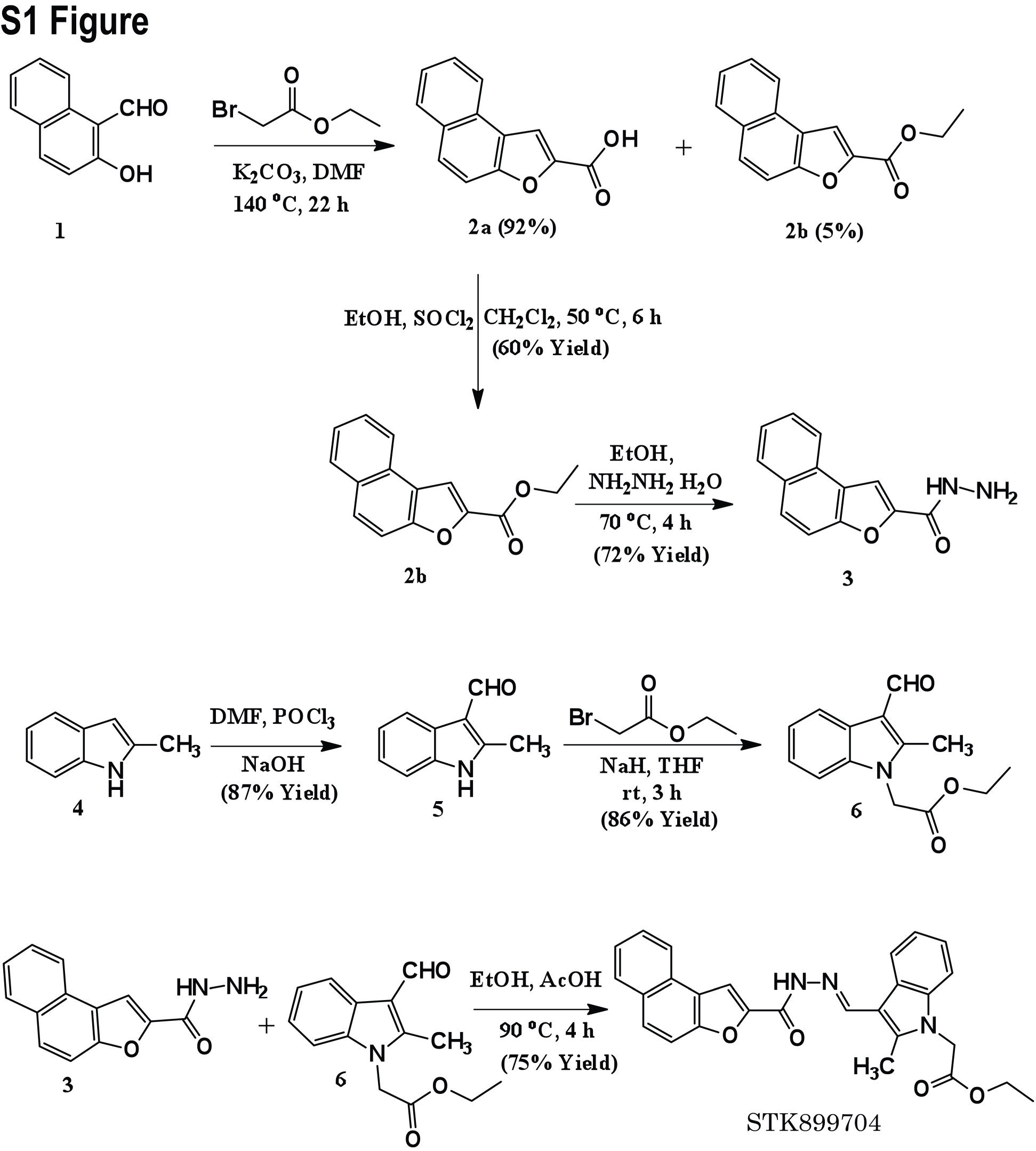

Supplement: S1 Fig — Synthesis of (E)-ethyl 2-(2-methyl-3-((2-(naphtho[2,1-b]furan-2-carbonyl)hydrazono)methyl)-1H-indol-1-yl)acetate (STK899704) is outlined in Scheme 1. The preparation of the title compound STK899704 was started from 2-Hydroxy-1-naphthaldehyde (1) and ethyl bromoacetate to obtaine Naphtho[2,1-b]furan-2-carboxylic acid (2a). This reaction required anhydrous K2CO3 as base and dimethylformamide (DMF) as solvent, both condensation as well as cyclization occurred in single step in 90% yield. The structure of the product 2a was determined by 1H NMR and ESIMS analyses. The 7 aromatic protons characteristic signals appeared between δ8.269–7.460 ppm in 1H NMR and 13 carbon signals appeared between δ 161.293–105.238 ppm in 13C NMR spectra, also its mass spectrum revealed a molecular ion peak at m/z 211.4[M-H]-corresponding to the molecular formula C13H8O3 confirms the structure of 2a. In the second step of reacton, Naphtho[2,1-b]furan-2-carboxylic acid (2a) converted to the corresponding ester ethyl naphtho[2,1-b]furan-2-carboxylate (2b) in 60% yield, by esterification reaction using ethanol and SOCl2. The carboxylate 2b was confirmed by the presence of new peaks quartet—CH2 at δ 4.424–4.370 ppm (J = 7.2 Hz) and triplet—CH3 at δ 1.388–1.353 ppm (J = 7.2 Hz) along with 7 aromatic protons in 1H NMR spectrum and also by molecular ion peak at m/z 241.4 [M+H]+, 263.4 [M+Na]+ appeared in ESIMS spectrum corresponding to the molecular formula C15H12O3.Thus compound 2b was reacted with hydrazine hydrate in third step of reaction to obtain an intermediate compound naphtho[2,1-b]furan-2-carbohydrazide 3 in 72% yield. 1H NMR spectrum of compound 3 exhibited no peak corresponds to ester instead it shows signals at δ 10.049 ppm for amide O = C-NH and δ 4.597 ppm for -NH2 (D2O exchangeable) of hydrazide respectively. The structure was further confirmed by recording its mass spectra, by its molecular ion peak at m/z 225.4 [M-H]-, 227.4 [M+H]+, 249.4 [M+Na]+corresponds to molecular formula C13H [file pone.0173311.s001.tif]

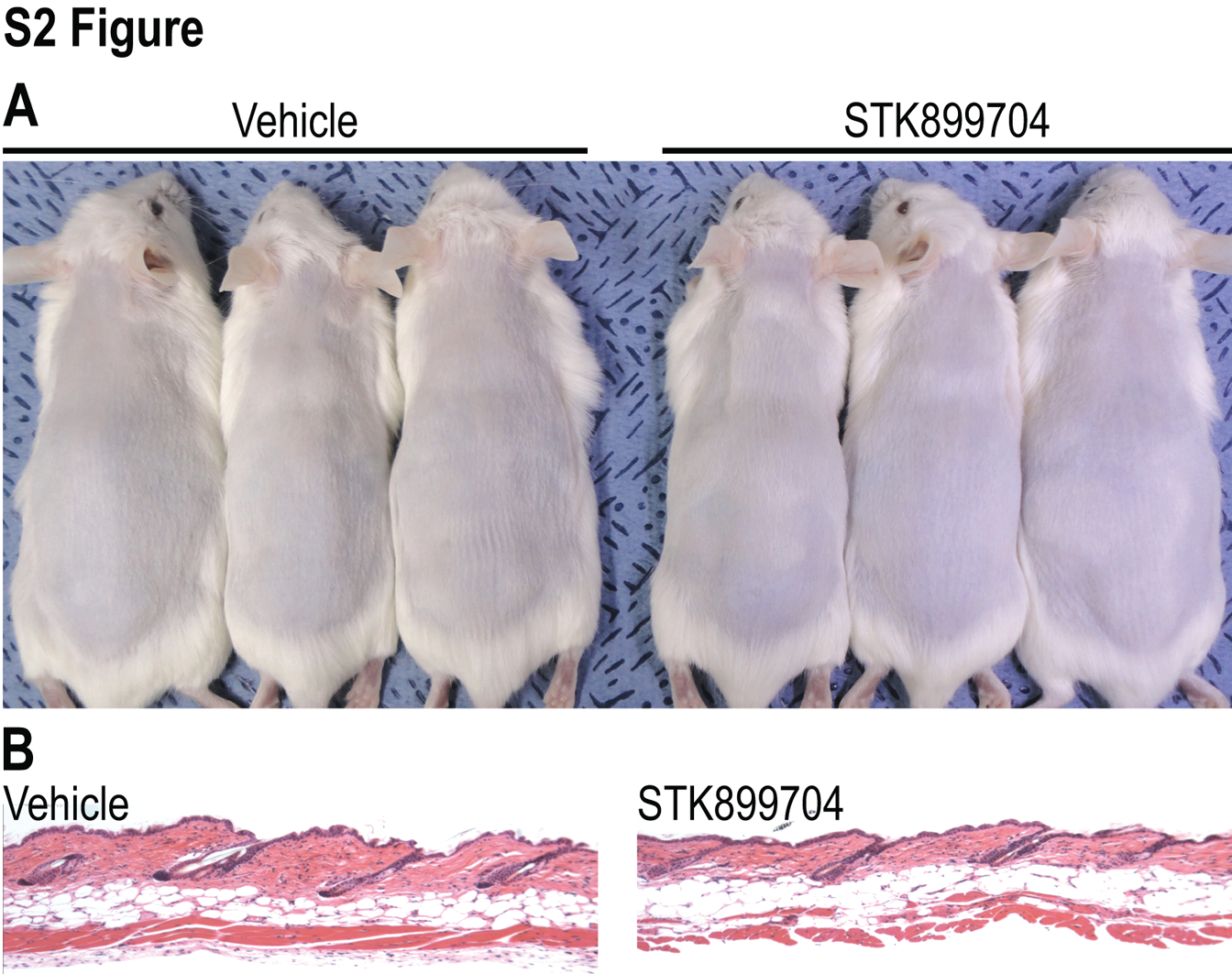

Supplement: S2 Fig — To examine additional effects of STK899704 treatment on normal skin, the compound or acetone was applied onto the dorsal skin of healthy mice twice weekly for 10 weeks. (A) The picture taken after the last treatment. (B) Hematoxylin/eosin stained sections from skin samples exhibit no skin abnormalities caused by STK899704 treatment. (TIF) [file pone.0173311.s002.tif]
