# Supplementary material for: Anticancer activity of a novel small molecule tubulin inhibitor STK899704
Source: PLoS One. 2017 Mar 15;12(3):e0173311. doi: 10.1371/journal.pone.0173311 (PMC5351965; doi:10.1371/journal.pone.0173311)
Supplement: S1 Table — The various cancer cell lines were cultured in microtiter plates (1–2 x 10^3^ cells/well) and incubated with different concentration of STK899704 for 4 days. The MTT assay was used to determine the cytotoxic effect of STK899704 and IC50s were assessed by log-dose-response curves. Data are the average of triplicate assays. (PDF) [file pone.0173311.s003.pdf]

Supplementary Table

S1 Table. Antiproliferative activity of STK899704 on various cancer cell lines

**Tumor**	**Cell line**	**IC_{50} (µM)**
Cervical carcinoma	HeLa	0.35
Epidermoid carcinoma	A431	0.48
Osteosarcoma	U-2OS	0.60
Breast carcinoma	MCF7	1.54
	MDA-MB-231	1.10
Colon carcinoma	HCT-116	0.73
	HT-29	0.71
Prostate carcinoma	PC-3	0.67
Lung carcinoma	A549	0.84
	NCI-H460	0.94
Gastric carcinoma	SNU-484	0.37
	SNU-601	0.92
Glioblastoma	A-172	0.64
	SNB-75	0.61
	U-373 MG	0.49
Hepatocellular carcinoma	HepG2	1.02
	Hep3B	0.69
Leukemia	HL-60	0.48
	K562	0.48

The various cancer cell lines were cultured in microtiter plates (1-2 x 10^3 cells/well) and incubated with different concentration of STK899704 for 4 days. The MTT assay was used to determine the cytotoxic effect of STK899704 and IC_{50}s were assessed by log-dose-response curves. Data are the average of triplicate assays.
